# Efficacy of Elaeagnus angustifolia Topical Gel in the Treatment of Symptomatic Oral Lichen Planus

**DOI:** 10.5681/joddd.2010.008

**Published:** 2010-03-14

**Authors:** Jamileh Beigom Taheri, Fahimeh Anbari, Ziba Maleki, Shiva Boostani, Afshin Zarghi, Firoz Pouralibaba

**Affiliations:** ^1^ Associate Professor, Department of Oral Medicine, Faculty of Dentistry, Shahid Beheshti University of Medical Sciences, Tehran, Iran; ^2^ Post-graduate Student, Department of Oral Medicine, Faculty of Dentistry, Shahid Beheshti University of Medical Sciences, Tehran, Iran; ^3^ Professor, Department of Oral Medicine, Faculty of Dentistry, Shahid Beheshti University of Medical Sciences, Tehran, Iran; ^4^ Oral Medicine Specialist, Private Practice, Tehran, Iran; ^5^ Professor, Department of Pharmaceutical Chemistry, Faculty of Pharmacy, Shahid Beheshti University of Medical Sciences, Tehran, Iran; ^6^ Assistant Professor, Department of Oral Medicine, Faculty of Dentistry, Tabriz University of Medical Sciences, Tabriz, Iran

**Keywords:** Elaeagnus angustifolia, lichen planus, management

## Abstract

**Background and aims:**

The purpose of this study was to determine efficacy of 19% Elaeagnus angustifolia (EA) topical gel in the treatment of symptomatic oral lichen planus.

**Materials and methods:**

Patients with symptomatic oral lichen planus referring to the Department of Oral Medicine, Fac-ulty of Dentistry at Shahid Beheshti University of Medical Sciences were asked to participate in the study. Twenty-eight pa-tients who were histopathologically diagnosed with lichen planus were divided into two groups (15 in the case and 13 in the control groups). The subjects were randomly assigned to either topical gel of EA or placebo in a double-blind manner. They were then instructed to apply the medication on dried lesions three times daily. Pain and size of the lesions were evaluated after 2 weeks. Data were analyzed by SPSS 12.0 software, using t-test, paired t-test, Fisher’s exact test and chi-square test.

**Results:**

Twenty-eight patients (m/f: 7/21) with symptomatic oral lichen planus participated in the study. Fifteen patients (m/f: 4/11) received E A gel and 13 patients (m/f: 3/10) received placebo. There was a 75% decrease in pain (33.3% in the case and 7.7% in the control groups), and a decrease of 50% in size (33.3% in the case group) and 75% only in 7.6% of the case group.

**Conclusion:**

The results suggest that 19% EA gel is efficient in the treatment of symptomatic oral lichen planus, with anti-inflammatory and analgesic effects, as well.

## Introduction

Oral lichen planus (OLP) is a chronic mucocutaneous disease with various clinical forms (reticular, papular, plaque-like, bullous, ulcerative).^1 - 3^ In the literature, different prevalence rates have been reported for OLP, varying from 2.2 to 15%.Typically, the reticular, papular and plaque-like forms of OLP are asymptomatic. Bullous form is very unusual and ulcerative lesions are the most debilitating; patients complain of prickling sensation during food intake.^4^

The treatment of OLP is disappointing and controversial.^3^ All current treatment strategies are aiming at reducing or eliminating its symptoms. The most frequently described therapy for OLP has been the administration of topical or systemic corticosteroids.^4^ Despite the therapeutic effects of topical corticosteroids, they have significant morbidity and disturbing adverse effects such as fungal infections and adrenal suppression, resulting in a continuing search for novel therapies.^5 - 7^

Since the ancient times, herbal drugs have been widely used for the treatment of diseases. Because of their negligible adverse effects there is an increasing tendency toward the use of herbal drugs worldwide.^8^ Elaeagnus angustifolia (EA) is one of these herbs with anti-inflammatory and analgesic effects. It also contributes to healing of the wounds and scar formations.^9^ The purpose of this randomized double-blind clinical trial was to evaluate the efficacy of 19% topical EA gel on treatment of symptomatic OLP.

## Materials and methods

Twenty-eight patients (M/F: 7/21; mean age: 48) who had been referred to the Department of Oral Medicine, Shahid Beheshti University of Medical Sciences, with a presumptive diagnosis of OLP were reexamined by an experienced clinician. After precise clinical evaluation to confirm OLP diagnosis a biopsy was taken for histopathologic evaluation.

Patients with any kind of systemic disease and those with a history of systemic corticosteroid use at least up to 2 months before were excluded from the study. Others were properly informed about the therapeutic aspects of the experimental approach. After their consent the patients were included in the study.

Lesion dimensions were measured by means of a transparent paper and duplicating it on a graph paper.

To prepare the desired formulation of aqueous extract of the EA, the fleshy part of its fruit was concentrated to reach a honey-like consistency. Carbapol P934, an edible polymer, was used as the base for formulation. The final product was 19% EA topical gel.

Patients were randomly divided into two groups. The EA gel was used on 15 patients in the test group and a placebo was prescribed for the 13 patients in the control group. Both drugs were administrated three times a day.

The patients were advised to brush their teeth and wash their mouth thoroughly after meal and apply the drug to the lesion site 45 minutes later, so that there would not be any possible interference between the drug and food ingredients. They were also advised to abstain from eating 30-60 minutes after using the drug. They were re-examined 14 days later.

Pain and extent of the lesions were evaluated. Pain was evaluated by asking the subjects to rank the severity of their pain on a visual analogue scale.

Data were analyzed with SPSS 12 software. To compare data collected before and after treatment in the same group, and to compare data in the two groups at different intervals, mixed-type variance analysis was used. Chi-square test and Fischer’s exact test were used for qualitative variables. A P value of < 0.05 was considered significant.

## Results

Data regarding evaluation of two principal parameters (pain and size) are displayed in Tables 1 and 2. Plaque index did not reveal any significant differences between the subjects.

**Table 1:** Pain severity before and after treatment and its changes in the case and control groups

Descriptive statistics					Statistical comparison				
					Equality of variances			Equality of means	
		Mean ± SD	Median	Range	F	p	t	df	P
Pre-treatment	Case Control	6.3 ± 1.5 6.2 ± 1.2	6 6	4-10 4-10	0.031	0.861	0.062	26	0.951
Post-treatment	Case Control	2.3 ± 1.8 4.2 ± 1.6	2 4	0-6 2-7	0.039	0.846	3.047	26	0.005
Reduction Amount	Case Control	4 ± 2.6 2 ± 2.2	4 2	(-1) - 9 (-1) - 6	0.283	00.599	2.183	26	0.038
Reduction percentage	Case Control	60.74 ± 33.19 29.25 ± 27.98	65.5 33.3	(-20)-100 (-16.7)-75	0.001	0.979	2.689	26	0.012

**Table 2:** Lesion size (the greatest dimension in cm) before and after treatment and its changes in the case and control groups

Descriptive statistics					Statistical comparison				
					Equality of variances			Equality of means	
		Mean ± SD	Median	Range	F	p	t	df	P
Pre-treatment	Case Control	2.2 ± 1.1 2.4 ± 0.8	2.2 2.3	0.6 - 4.2 1.2 - 3.6	1.885	0.182	0.379	26	0.707
Post-treatment	Case Control	1.5 ± 1 2.3 ± 0.8	1.5 2.3	0.0- 3 1 – 3.7	2.349	0.137	2.314	26	0.029
Reduction Amount	Case Control	0.8 ± 0.6 0.1 ± 0.1	0.5 0.0	0.1 – 1.5 (0.1) – 0.3	64.749	< 0.001	4.517	15.7	< 0.001
Reduction percentage	Case Control	37.24 ± 26.7 4.10 ± 7.39	33.3 0.0	4.6 – 100.3 (-4.76) – 17.7	10.066	0.004	4.607	16.4	< 0.001

Pain severity and its percentage decreased in the case group compared to the control group. According to variance analysis pain decreased generally subsequent to treatment (P < 0.001), with significant differences between the case group and control groups (P = 0.038). Considering paired t-test, the mean reduction of pain in the case group on the VAS was 4 cm. (P < 0.001). In the control group this reduction was 2 cm on VAS (P = 0.006)

In each group one person (6.7% in the case and 7.7% in the control groups) experienced more severe pain after intervention. One patient in the case group (6.7%) and three patients in the control group (23.1%) reported no change in their pain severity. Complete resolution of pain was reported only by two patients (13.3%) in the case group. Thirteen patients in the case group (86.7%) had less pain after treatment compared to 4 patients (30.8%) in the control group (P = 0.003).

According to variance analysis, size of the lesion decreased (P < 0.001) after treatment, with significant differences between the two groups (P < 0.001).

Size of the lesions in the case group minimized to about 0.7 cm on VAS (P < 0.001, paired t-test). In the control group size of the lesions decreased to about 0.7 cm, too, but this was not statistically significant (P < 0.82). In this group, at the end of the study two patients (15.4%) had larger lesions and in five patients (38.5%) the lesions remained unchanged in size. None of these findings were seen in the case group. Complete resolution was seen only in one patient (67%) in the case group.

## Discussion

EA is a herb of Elaeagnaceae family with various therapeutic effects. The dried fruit has the ability to accelerate wound healing. It is an antipyretic herb and can resolve headache as well. EA contains vitamins A and B and also vitamin K, which is effective in coagulation. In traditional medicine it is known as a wound healing accelerator and there are reports of its anti-inflammatory and analgesic effects because of its flavonoid terpenoid materials.^10 - 14^

EA healing effects have been confirmed on rats. Pharmacologists investigated anti-inflammatory and analgesic properties of EA on rats and its effects on osteoarthritis and the results were satisfactory.^12^ However, to date no studies have been carried out on the effects of EA on OLP, which is a chronic mucocutaneous disease with no known cure at present. Prevalence of OLP is estimated to be 0.2–4%. This condition afflicts middle-aged women more than others.^15 - 19^

Various drugs have been used to treat symptomatic OLP, including topical corticosteroids with or without systemic adjuvants. Immunosuppressive agents such as azathioperine, cyclosporine, etc have also been used in severe cases. These drugs have considerable side effects like candidiasis, renal toxicity, adrenal suppression, malignancy, headaches^19 - 22^ and transient burning sensation of mouth.^23^ Due to these adverse effects, researchers are trying to find a substitute for current treatment, especially in patients who cannot take corticosteroids.

In this study, we explored the efficacy of a new formulation of EA as 19% water-based gel in the topical treatment of symptomatic OLP and its effect was compared to a placebo. Before treatment there was no statistically significant difference in pain between the two groups. After applying our new formulation for 4 weeks, pain was less severe in the case group compared to the control group, with statistically significant differences. Success (i.e. at least 75% decrease in pain severity) was seen in 5 patients in the case group and only in one patient (7.7%) in the control group (P = 0.173).

Prior to treatment there were no statistically significant differences in the biggest dimension of the lesions between the two groups. However, post-treatment size of lesions in the case group was smaller than that in the control group. Success (i.e. a decrease in lesion extent to half of its original size) was noted in just 5 patients in the case group (33.3%), which was statistically significant (P = 0.044).

## Conclusion

Our results showed that Elaeagnus angustifolia used topically in the form of 19% aqueous gel has anti-inflammatory, analgesic and healing effects on symptomatic oral lichen planus.
